# Contrasting diversity patterns of brown: and white-rot wood saprotrophs in response to climate and dispersal vectors

**DOI:** 10.1093/femsec/fiaf116

**Published:** 2025-11-21

**Authors:** Anika Gossmann, Kadri Runnel, Mohammad Bahram, Thomas Ranius

**Affiliations:** Swedish University of Agricultural Sciences, Department of Ecology, Uppsala, 75651, Sweden; University of Tartu, Faculty of Science and Technology, Tartu, 50090, Estonia; Swedish University of Agricultural Sciences, Department of Ecology, Uppsala, 75651, Sweden; Aarhus University, Department of Agroecology, Slagelse, 4200, Denmark; Swedish University of Agricultural Sciences, Department of Ecology, Uppsala, 75651, Sweden

**Keywords:** dead wood, fungal diversity, hydroclimatic compensation model, latitudinal diversity gradient, microclimate, wood-inhabiting fungi

## Abstract

Climate is a major determinant of fungal diversity on both large and small spatial scales. However, little is known about the combined effects of regional temperature, microclimate, and dispersal vectors on fungal diversity. We studied the effect of microclimate and wood-inhabiting beetles serving as potential dispersal vectors on the diversity of wood-inhabiting fungi in general—and of brown- and white-rot fungi in particular—along a regional temperature gradient. This focus is motivated by the critical role that different rot types play in wood decomposition and carbon cycling. Beetle and fungal communities were sampled in 243 logs of Norway spruce (*Picea abies*), which were placed along a 1200 km latitudinal gradient in Sweden (i.e. regional temperature gradient) and under different shading conditions (i.e. microclimatic gradient). Species richness of brown-rot fungi increased with beetle abundance in both the south and the north, whereas shade level markedly limited their species richness only in the north. In contrast, white-rot fungi were unaffected by either factor. These findings highlight that fungal responses to microclimate and dispersal vectors may differ between regions and suggest that species richness of brown-rot fungi may increase with a warming climate, especially in the north.

## Teaser

Brown–and white–rot producing saprotrophs–fungi, which are of high importance for the decomposition process–show contrasting biodiversity patterns in their response to microclimate and dispersal vectors along a regional climatic gradient.

## Introduction

Understanding the main drivers of biodiversity patterns is a central question in ecology. Climate is one of the most important abiotic factors shaping species communities across various spatial scales (Thomas et al. 2011, Lee-Yaw et al. 2016). Generally, biodiversity increases with rising temperatures along regional temperature gradients, generating a latitudinal diversity gradient (Pianka 1966). This aligns with the energy-richness hypothesis, which argues that higher productivity (conversion of energy into biomass) in warmer regions leads to higher species diversity (Rosenzweig and Abramsky 1993). This trend has been observed for mammals (Buckley et al. 2010), birds (Hawkins et al. 2003), and insects (Goßmann et al. 2024). Fungi have also been shown to follow a latitudinal diversity gradient (Tedersoo et al. 2014, Mikryukov et al. 2023, Abrego et al. 2024), although this pattern is not always obtained (Větrovský et al. 2019, Li et al. 2023).

Fungi are one of the most diverse groups of organisms on Earth, with many species inhabiting wood (Niskanen et al. 2023). Among fungi that colonize wood, saprotrophs—those that decompose wood—are the most functionally important, and can be divided into brown- and white-rot fungi. The latter employ a more efficient wood decay strategy by decomposing all wood components (lignin, cellulose, and hemicellulose), whereas brown-rotters decompose only cellulose and hemicellulose (Highley and Kirk 1979). The dominance of either of these two groups can have large ecosystem effects, influencing carbon sequestration in forest ecosystems, tree seedling regeneration, and communities of dead-wood inhabiting species (Fukasawa 2021).

Along large-scale climatic gradients, it has been shown that species richness of saprotrophic fungi increases and their community composition alters toward warmer regions (Tedersoo et al. 2014). At the same time, microclimate is an important factor driving the diversity and function of wood-inhabiting fungi on smaller spatial scales (Gilbertson 1980, Rayner and Boddy 1988, Lindhe et al. 2004, Bradford et al. 2014). The diversity of wood-inhabiting fungi in general has been shown to be higher under closed canopies (Bässler et al. 2010, Thorn et al. 2018), which provide cooler and more humid microclimatic conditions. In contrast, the group of brown-rotters benefits from open conditions with higher temperatures and low humidity (Fukasawa 2021, Brabcová et al. 2022). It has been suggested that brown-rotters have adapted to stressful conditions like those found in sun-exposed dead wood due to lower competitive pressure in these environments (Fukasawa 2021).

According to the “hydroclimatic compensation model”, species’ habitat requirements can vary along regional climatic gradients (Ackerly et al. 2020). Thus, to compensate for regional temperature, species might occur in warmer habitats at their cold-range margins, and in cooler habitats at their warm-range margin. There is empirical support for this model from studies of lichens, vascular plants, and insects (Christiansen et al. 2022, Lõhmus et al. 2023, Goßmann et al. 2024), but to our knowledge not on fungi. Thus, it remains to be studied whether the habitat requirements of the different fungal groups, such as brown- and white-rotters, may vary along a regional climatic gradient, supporting the “hydroclimatic compensation model” (Ackerly et al. 2020).

The latitudinal diversity gradient may directly result from organisms benefiting from warmer conditions (Rosenzweig and Abramsky 1993). Moreover, it can be derived from increased species richness and abundance of host plants, prey, or other resources favored by warmer conditions. For wood-inhabiting fungi, beetles transporting fungal spores could serve as one of such resources (Birkemoe et al. 2018), shaping fungal communities in wood (Persson et al. 2011, Strid et al. 2014, Seibold et al. 2019). For instance, the spruce bark beetle *Ips typographus* (L.) has been observed to vector over 30 fungal species (Solheim 1993). While beetles clearly contribute to fungal dispersal, it remains unclear whether they influence diversity patterns of fungi in dead wood in general and, in particular, the functionally distinct groups of brown- and white-rot fungi. In colder regions, insects are active for shorter periods and occur in reduced numbers (Lindman et al. 2023). As a result, their potential to act as fungal dispersal vectors may be limited. In contrast, in warmer regions, sun-exposed dead wood preferred by beetles can become too dry and warm for fungal establishment (Norros et al. 2015). Thus, the relative importance of dispersal vectors and microclimate may differ along a regional temperature gradient for wood-inhabiting fungi.

In this study, we tested predictions based on the latitudinal diversity gradient and the “hydroclimatic compensation model” on wood-inhabiting fungi. We determined diversity distribution patterns of fungi in general, and brown- and white-rot producing saprotrophs in particular, along regional temperature and microclimatic gradients, and in relation to beetle vectors. For this, we placed 243 logs of Norway spruce under contrasting shading conditions, creating a small-scale microclimatic gradient along a 1200 km latitudinal gradient in Sweden. We predicted the following:

- Wood-inhabiting fungal richness, including brown-rot and white-rot fungi, increases with regional temperature, following a latitudinal diversity gradient. As white-rot fungi are favored by cool and humid microclimates (Fukasawa et al. 2025), we expect their fungal richness and relative abundance to be higher in shaded logs. In contrast, since brown-rot fungi benefit from warmer microclimates (Fukasawa et al. 2025), we predict higher fungal richness and relative abundance in sun-exposed logs. Lastly, we expect the community composition of all wood-inhabiting fungi, and brown- and white-rotters to be more strongly structured along the regional temperature gradient than along the shade gradient due to larger dispersal distances.
- Saproxylic beetles serve as dispersal vectors for wood-inhabiting fungi (Birkemoe et al. 2018). Therefore, we expect their abundance to explain further variation in fungal richness, relative abundance and community composition.
- Following the “hydroclimatic compensation model”, shade level is a more limiting factor than dispersal vectors for wood-inhabiting fungi in warmer regions, as sun-exposed conditions may be less favorable. In contrast, in colder regions, dispersal vectors may be a more limiting factor than shade level due to reduced insect activity.

## Material and methods

### Study regions and study design

**Figure 1.**
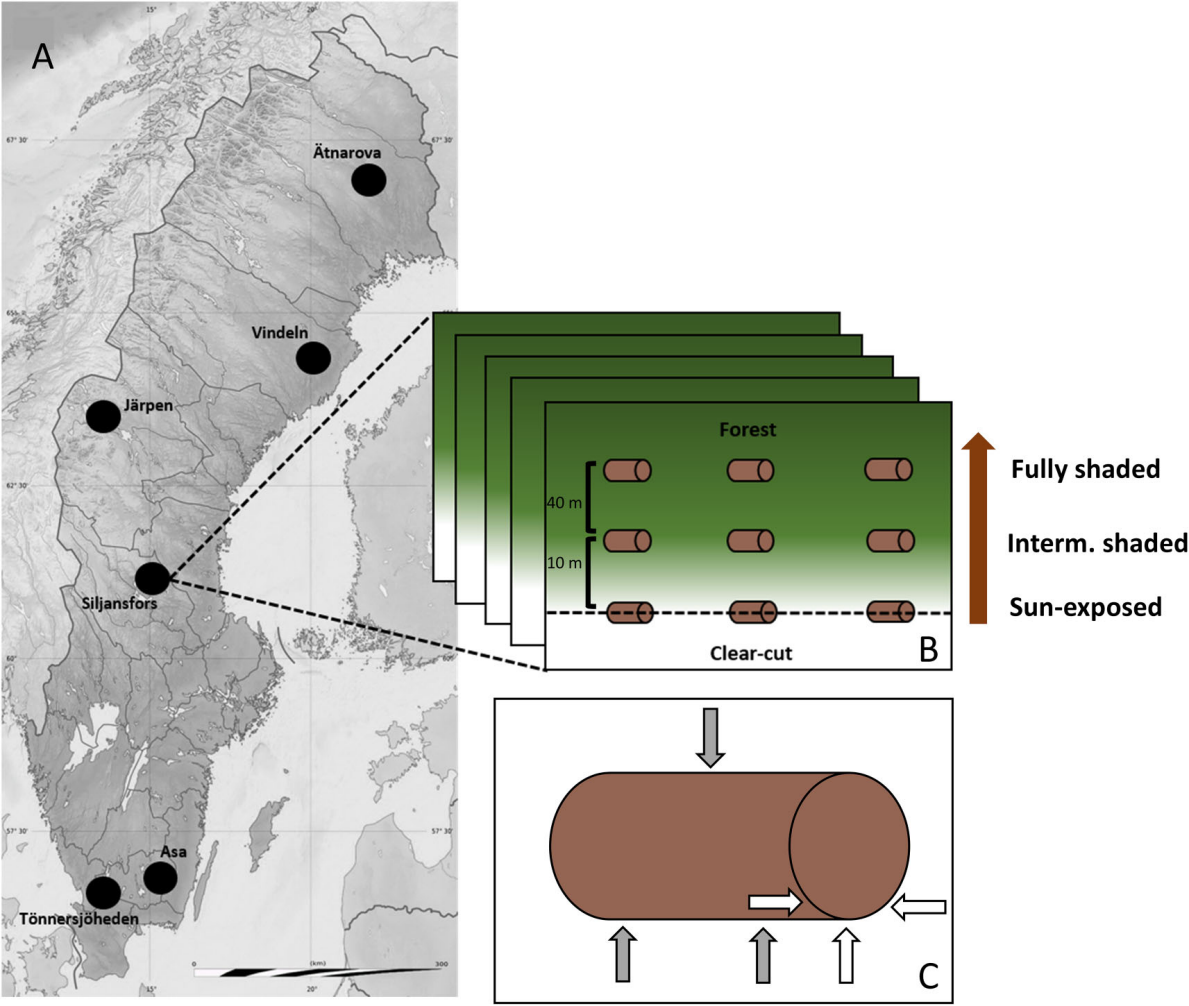
Figure adapted from Goßmann et al. (2024). Six study regions, which consist of five study plots each (except Siljansfors with three, and Vindeln with four), were distributed over Sweden (a). A plot consists of 9 stem sections on three different shade levels: (i) sun-exposed, (ii) intermediately shaded, and (iii) fully shaded. The dashed line represents the forest edge (b). Samples were collected by drilling into each spruce log, with three holes for the first sample type (grey arrows), and three holes for the second sample type (white arrows) (c).

We selected six study regions along a 1200 km latitudinal gradient in Sweden, where we established five study plots each (Fig. 1a). In two of the regions (Siljansfors and Vindeln), forestry operations later destroyed some plots, so these regions were included with three and four plots, respectively. The study regions are dominated by managed forests of Norway spruce (*Picea abies* L.) and Scots pine (*Pinus sylvestris*. L.), with the 30-year mean annual temperatures (MAT) ranging between 7.8°C in the south to 0.3°C in the north (1991–2020; SMHI, 2022). The monthly average precipitation ranged from 76.6 mm at the southernmost study region to 63.7 mm at the northernmost study region (1991–2020; SMHI, 2022). We selected study plots based on the criterion that they contained a mature spruce forest stand adjacent to a 1–2-year-old clear-cut. On average, the plots within a study region were 30 km apart (range: 2 km–102 km). In March and April 2020, we placed 9 spruce logs of 1.5 m length originating from the same tree along three shade levels at each study plot: (i) three logs at the forest edge between the clear-cut and the mature spruce stand, i.e. sun-exposed, (ii) three logs 10 m into the forest stand, i.e. intermediately shaded and (iii) three logs 50 m from the forest edge into the forest, i.e. fully shaded (Fig. 1b). This resulted in 243 experimental spruce logs in total. The diameter of the logs was 15–35 cm, and the distance between the logs at the same shade level was 15 m. From the beginning of May until mid-September 2020, we measured the microclimatic temperature at each shade level per study plot and confirmed that the mean microclimatic temperature decreased along the shade gradient from sun-exposed to fully shaded logs (details in Goßmann et al. 2024).

### Fungal sampling

The unit of analysis in this study was the fungal community at each shade level within each study plot (N = 81; 3 shade levels across 27 plots). To characterize these fungal communities across different parts of the logs, we combined data from two distinct sample sets collected in September 2023.

- The first set of samples aimed to provide a general representation of fungi inhabiting experimental logs at different shade levels. To obtain the samples, sawdust was collected from three holes drilled into each log: two on the underside, where the log was in contact with the ground, close to each end, and one on the top center (Fig. 1c). All holes were drilled perpendicularly to the log surface. Sawdust was not sampled on log level, but was directly pooled per plot and shade level, with one composite sample representing three logs per shade level, resulting in 81 samples (3 shade levels across 27 plots).
- The second set of samples aimed to characterize the fungal communities colonizing the cut ends of each experimental log, to be linked in a separate study to the decomposition rates of cut wood discs. For this, sawdust was collected from three holes drilled perpendicularly into the lower section of the larger cut end of each of the 243 logs (Fig. 1c). The sawdust was pooled at log level, resulting in 243 individual samples (9 logs in each of the 27 plots), kept separate until postsequencing.

Thus, each plot shade level was ultimately represented by four sequenced samples. To increase representativeness of data collected from each plot shade level, these two datasets were combined by calculating a weighted mean of OTU (Operational Taxonomic Unit) read counts, where the composite samples from the first set (each representing three logs) were given three times more weight than the cut-end samples from the second set (each representing one log), ensuring proportional representation of log-level contributions. To ensure that combining the two datasets does not introduce biases, we analyzed the two datasets also separately (Appendix S1).

The sampling technique was the same for both sample types: first, the bark was removed with an ethanol-flamed knife. Then, sawdust was collected in plastic bags by drilling into the experimental log using a 1 cm diameter drill bit to a depth of 10 cm. The extracted sawdust was freeze-dried at −50°C for 24 h and homogenized using a swing mill and plastic beads. Fungal DNA was extracted from 100 mg of each homogenized wood sample using the NucleoMag DNA Microbiome kit (Macherey-Nagel, Germany). Subsequently, DNA concentration and quality were measured for each sample before PCR. The fungal ITS (internal transcribed spacer) region was amplified by using the primers fITS7 (forward) (Ihrmark et al. 2012) and ITS4 (reverse) (White et al. 1990). PacBio sequencing was performed externally by SciLifeLab, Uppsala (Sweden).

### Bioinformatics

The raw sequences were processed (including demultiplexing, filtering, and dereplication) using the LotuS 2.32 pipeline (Özkurt et al. 2022). The pipeline was configured with the following settings: dereplication threshold (-derepMin 2), and clustering algorithm (Fu et al. 2012). From 6.8 million reads, 4.6 million high-quality reads retained, which were clustered into OTUs using a 98.5% similarity threshold. Both de novo and reference chimera filtering (via Uchime (Edgar 2016)) were applied, as well as ITSx (Bengtsson-Palme et al. 2013) to remove non-ITS OTUs, leaving 2817 OTUs. Taxonomy was assigned using the EUKARYOME reference database (Tedersoo et al. 2024) using BLAST. Fungal OTUs were functionally annotated at the genus level using the FungalTraits tool (Põlme et al. 2020). With “wood-inhabiting fungi”, we refer to all fungi we collected in the spruce logs.

### Beetle sampling

We sampled saproxylic beetles emerging from the spruce logs using eclector traps from April to September 2021 (Goßmann et al. 2024). We used the abundance (number of individuals) of sampled beetles as proxy for the abundance of adult beetles colonizing the logs one year before the sampling. Beetle abundance was dominated by the bark beetles *Crypturgus sp*. (3.8%)*, Pityogenes chalcographus* (68.2%), and *I. typographus* (10.0%).

### Statistics

Statistical analyses were conducted in R 4.2.0 (R Core Team, 2022). Sample coverage (i.e. the proportion of the total community represented by the observed species; Chao and Jost 2012) was consistently high across samples (mean ± SD = 0.995 ± 0.003). Beta regression (*betareg* function from package *betareg*, Cribari-Neto, and Zeileis 2010) revealed no significant effects of regional temperature or shade level on coverage, and only a weak negative relationship with beetle abundance (Est. = −0.04; *P* = 0.001). For the following analyses, we rarified the dataset onto the median read count per sample.

To test the effects of regional temperature, shade level and beetle vectors on fungal richness (predictions I and II), we applied generalized linear mixed-effects models (*glmmTMB* from package *glmmTMB*, Brooks et al. 2017) with OTU richness of all fungi combined, brown-rotters and white-rotters per plot shade level as response variable. A negative binomial error distribution was used in the models, as the Poisson distribution (commonly applied to count data) showed overdispersion. Predictor variables included regional temperature, shade level, their interaction, and log-transformed beetle abundance. We checked for multicollinearity among predictor variables with the function *check_collinearity* from the performance package (Lüdecke et al. 2019). The random effects structure was defined as plot nested within study region (*Study_region/Plot*). Regional temperature referred to the 30-year mean annual temperature (SMHI, 1991–2020). A Wald Chi-square test was used to estimate the statistical significance of the predictor variables.

Similar models were built with the relative abundance of brown- and white-rotters (i.e. the proportion of sequencing reads belonging to a specific functional group relative to all sequencing reads) as response variables. We used a Tweedie distribution when modeling brown-rot relative abundance as the response variable, and a Beta distribution when modeling white-rot relative abundance.

To analyze how the community composition of all fungi combined, and brown- and white-rotters varies along a regional temperature and a shade gradient, we applied a permutational multivariate analysis of variance with Bray–Curtis dissimilarity matrices (PERMANOVA, function *adonis* from R package *vegan*; Oksanen 2010), using rarefied abundances of those OTUs that occurred in more than one sample. Regional temperature, shade level and beetle abundance were used as predictor variables. To account for the experimental design, we included a strata variable to constrain permutations within study regions. The strata were defined by a factor variable *Studyregion_Plot*.

Lastly, to test whether the relative importance of shade level and dispersal vectors varies along a regional temperature gradient (prediction III), we categorized the dataset into southern (Asa, Tönnersjöheden, and Siljansfors) and northern regions (Järpen, Vindeln, and Ätnarova). We chose this approach instead of including an interaction term between regional climate and beetle abundance, since these variables are correlated (Pearson correlation, cor = 0.27, *P* = 0.01) leading to different coefficient directions dependent on whether the interaction term was included or not. For the southern and the northern regions, we applied the same generalized linear mixed-effects models as for the total data set including shade level, and beetle abundance as predictor variables. Subsequently, to determine whether beetle abundance or shade level better explains the observed pattern, we calculated the ∆AIC, representing the change in AIC when a variable is excluded from the full model.

## Results

Across all study regions, we sampled 2417 wood-inhabiting fungal species (OTUs) in experimental spruce logs. Of these, 1107 could be identified to genus level, and thus, be categorized into different functional groups, with 40 brown-rot species and 122 white-rot species.

### Effects of regional temperature, shade level, and dispersal vectors on fungal diversity

Richness of all fungi combined was not affected by any of the tested variables (i.e. regional temperature, shade level, interaction between shade level and regional temperature, and beetle abundance; Table 1, S2).

**Table 1.** Wald Chi-square test of generalized linear mixed model with species richness of all fungi, and brown- and white-rotters as response variables.

Model with predictor variables	Marginal and conditional R*^2^*	*χ* ^2^	DF	*P*-value
**All fungi**	0.16; 0.41			
Shade level		2.73	2	0.26
Regional climate		2.69	1	0.1
Beetle abundance		2.13	1	0.14
Regional climate: Shade level		0.46	2	0.79
**Brown-rotters**	0.23; 0.39			
Shade level		4.48	2	0.11
Regional climate		1.71	1	0.19
Beetle abundance		10.5	1	**0.001**
Regional climate: Shade level		4.71	2	*0.09*
**White-rotters**	0.06; 0.55			
Shade level		0.1	2	0.95
Regional climate		0.67	1	0.41
Beetle abundance		1.05	1	0.31
Regional climate: Shade level		2.43	2	0.29

Numbers in bold indicate a significant effect (<0.05), numbers in italic indicate a marginal significant effect (<0.1). The marginal R^2^ refers to the measured proportion of variance explained only by the fixed effects, whereas the conditional R^2^ accounts for both fixed and random effects.

Richness and relative abundance of brown-rot fungi increased significantly with beetle abundance. Relative abundance of brown-rotters was significantly higher in sun-exposed than shaded logs, but their richness did not differ between the shade levels. Regional temperature and its interaction with shade level had no effect on either richness or relative abundance of brown-rotters (Table 1, 2, and S4).

**Table 2.** Wald Chi-square test of generalized linear mixed models with relative abundance of brown- and white-rotters as response variables.

Model with predictor variables	Marginal and conditional R*^2^*	*χ* ^2^	DF	*P*-value
**Brown-rotters**	1; 1			
Shade level		14.2	2	**<0.001**
Regional climate		1.27	1	0.26
Beetle abundance		4.39	1	**0.04**
Regional climate: Shade level		1.27	2	0.53
**White-rotters**	0.06; 0.06			
Shade level		0.59	2	0.74
Regional climate		0.56	1	0.46
Beetle abundance		0.44	1	0.51
Regional climate: Shade level		0.48	2	0.79

Numbers in bold indicate a significant effect (<0.05), numbers in italic indicate a marginal significant effect (<0.1). The marginal R^2^ refers to the measured proportion of variance explained only by the fixed effects, whereas the conditional R^2^ accounts for both fixed and random effects.

Richness and relative abundance of white-rotters were not affected by any of the tested variables (Table 1 and 2, S4).

### Effects of regional temperature, shade level, and dispersal vectors on fungal community composition

The community composition of all fungi combined changed marginally with regional temperature (Table S5). Along the shade gradient, we found a change in community composition of all fungi combined and brown-rotters (Table S5). Beetle abundance had no effect on the community composition of any tested fungal group (Table S5). Community composition of white-rot fungi were not affected by any of the tested variables (Table S5).

### Effect of dispersal vectors and shade level in northern and southern regions

**Figure 2.**
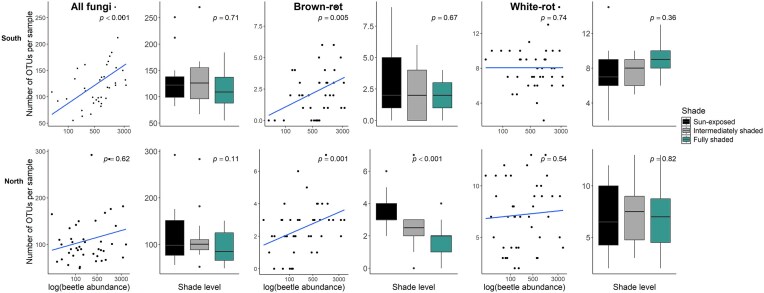
Species richness in spruce logs of all fungi, brown-rotters, and white-rotters along a beetle abundance gradient (log-transformed), and along a shade gradient (respective boxplots to the right). The upper panels show the effects in the south (Tönnersjöheden, Asa, Siljansfors), and the lower panels the effects in the north (Vindeln, Järpen, Ätnarova).

In the southern study regions, richness of all fungi inhabiting spruce logs increased significantly with beetle abundance, while shade level had no effect. In the north, neither beetle abundance nor shade level affected richness of all fungi (Fig. 2, Table 3a, S6).

**Table 3.** Wald Chi-square test of generalized linear mixed model with species richness of wood-inhabiting fungi, and brown- and white-rotters as response variables.

Model with predictor variables	AICc	∆AICc	Marginal / conditional R^2^	*χ* ^2^	DF	*P*-value
**South**						
**All fungi**	406.9		0.31 / 0.42			
Shade level		−5.1		0.69	2	0.71
Beetle abundance		+4.5		11.9	1	**<0.001**
**Brown-rotters**	159.4		0.29/ 0.63			
Shade level		−4.6		0.79	2	0.67
Beetle abundance		+6.9		7.86	1	**0.005**
**White-rotters**	193.8		0.04 / 0.51			
Shade level		−3.9		2.08	2	0.36
Beetle abundance		−2.9		0.11	1	0.74
**North**						
**All fungi**	437.4		0.05/ 0.55			
Shade level		−1.8		4.38	2	0.11
Beetle abundance		−2.7		0.24	1	0.62
**Brown-rotters**	141.4		0.39/ 0.58			
Shade level		+8.8		18.6	2	**<0.001**
Beetle abundance		+6.2		10.3	1	**0.001**
**White-rotters**	233.7		0.01 / 0.37			
Shade level		−4.9		0.41	2	0.82
Beetle abundance		−2.4		0.38	1	0.54

The dataset was separated into south (Tönnersjöheden, Asa, Siljansfors) and north (Vindeln, Järpen, Ätnarova). Numbers in bold indicate a significant effect (< 0.05), numbers in italic indicate a marginal significant effect (< 0.1). To determine whether beetle abundance or shade level better explains the observed pattern, we calculated the ∆AICc, representing the change in AICc when each variable is excluded from the full model. The marginal R^2^ refers to the measured proportion of variance explained only by the fixed effects, whereas the conditional R^2^ accounts for both fixed and random effects.

Richness of brown-rot fungi increased with beetle abundance in both regions. However, shade level had a strong effect in the north, where richness was considerably higher in sun-exposed than in shaded logs (Fig. 2, Table 3, S6). Based on ∆AIC value, shade level was a better predictor of brown-rot richness than beetle abundance in the north (Fig. 2, Table 3). This suggests that dispersal vectors may have been more limiting in the south, while microclimatic conditions were more limiting in the north.

For the richness of white-rotters, we did not detect any effect of shade level or beetle abundance in either the southern or in the northern study region (Fig. 2, Table 3, S6).

### Comparison of datasets

While our central approach was to perform analyses on data combining metabarcoding results from two datasets (one collected from the cut ends and the other along the experimental logs), we also applied the same analyses on the datasets separately to check the robustness of the results. The outcomes were largely consistent with those presented above (Appendix S1).

## Discussion

Our study revealed clear differences in how environmental drivers shape the diversity of brown- and white-rot fungi. Brown-rot fungi responded most strongly to both dispersal vectors and microclimate: their richness increased with beetle abundance across all regions, and in the north, was additionally affected by shade level. In contrast, richness of white-rot fungi showed no response to any of the tested variables. These findings highlight distinct ecological strategies between the two functional groups and suggest that their diversity may respond to environmental changes in fundamentally different ways.

### Fungal diversity along a regional temperature and microclimatic gradient

We did not find support for a latitudinal diversity gradient of wood-inhabiting fungi. On a global scale, previous studies have reported such patterns for soil fungi (Bahram et al. 2018, Mikryukov et al. 2023), whereas other studies have found the opposite trend, with soil fungal richness decreasing toward warmer regions (Větrovský et al. 2019, Li et al. 2023). Regarding wood-inhabiting fungi, Englmeier et al. (2023) detected an increasing fungal richness with regional temperature, but this study did not cover a large temperature gradient. Taken together with our results, this suggests that fungi may not follow latitudinal diversity gradients as consistently as other taxonomical groups, such as mammals (Buckley et al. 2010), birds (Hawkins et al. 2003), or insects (Müller et al. 2015).

The relative abundance of brown-rotters increased from shaded to sun-exposed logs, whereas all fungi combined and white-rot fungi were not affected by shade level. Previous studies revealed higher brown-rot diversity in warmer and drier microclimates, such as in sun-exposed dead wood (Fukasawa 2021, Brabcová et al. 2022). Such conditions can be stressful for fungi due to dryness, elevated temperatures, and radiation (Krah et al. 2021). Brown-rotters may be better adapted to these conditions (Fukasawa 2021) since they use a nonenzymatic oxidative system to initiate cellulose degradation. This cellulose degradation pathway is less dependent on sustained enzyme activity, oxidative stress and high moisture compared to white-rot fungi, whose lignin-degrading enzymes are more sensitive to desiccation and require moist conditions (Castaño et al. 2021). Thus, in sun-exposed wood, brown-rot fungi may outcompete white-rot fungi.

Community composition of all wood-inhabiting fungi changed only marginally along the regional climatic gradient but was significantly affected by shade level. The effect of shade level was also apparent for brown-rotters, a relatively small group among the whole fungal community inhabiting the experimental logs. A gradual change in fungal community composition on similar substrates with increasing geographical distance has been reported by several studies (e.g. Kubart et al. 2016, Abrego et al. 2018). Such a pattern has been explained by dispersal limitations and changing macro-environmental conditions (Chaudhary et al. 2022). However, our study suggests that microclimate may be a stronger determinant for fungal communities, causing greater compositional changes over short distances than large-scale environmental drivers over long distances.

### Dispersal vectors as important drivers for fungal diversity along a regional temperature gradient

We found positive relationships between brown-rot fungal diversity and beetle abundances, supporting earlier studies on beetle-mediated fungal dispersal (Jacobsen et al. 2017, Birkemoe et al. 2018, Seibold et al. 2019). Our results show that brown-rot fungal diversity—not just the occurrence of specific brown-rot species, like *Fomitopsis pinicola*—is enhanced by the abundance of beetle vectors (Persson et al. 2011, Pouska et al. 2013, Vogel et al. 2017). This may help explain why brown-rot fungi were most abundant in sun-exposed logs: not only are they physiologically suited to dry, sunlit wood, but they also benefit from increased colonization opportunities via beetles, which often prefer similarly warm and open conditions (Goßmann et al. 2024). Thus, beetle-facilitated dispersal and favorable microclimate may jointly reinforce brown-rot diversity in sun-exposed habitats.

We used the abundance of emerging beetles as a proxy for the abundance of adult beetles that colonized the logs one year prior to sampling. However, beetle emergence is also affected by their survival rates, which can be influenced by competition or various environmental factors (Brin and Bouget 2018). Therefore, we cannot rule out that an observed low emerging beetle abundance reflects a low survival rate, rather than a low abundance of beetles that initially colonized the wood. Thus, one reason for some weak or absent correlations between beetle abundance and fungal richness in our study could be that the proxy we used as dispersal vectors was not ideal. Assessing the abundance of saproxylic beetles entering dead wood items one year before fungal sampling may have served as a better proxy for dispersal vectors.

### Effect of shade level and dispersal vectors on wood-inhabiting fungi in southern and northern regions

We predicted that reduced abundance and activity in the north would result in beetles being a more limiting factor for fungal dispersal, whereas shade level might be a limiting factor in the south due to too warm and dry conditions in sun-exposed dead wood. However, our study revealed a positive effect of beetle abundance on richness of all fungi only in the south, whereas shade level had no effect. In contrast, species richness of brown-rotters was positively affected by beetle abundance in both the south and the north, but shade level had an effect only in the north. The positive relationship between beetle abundance and richness of all fungi might be due to higher beetle abundances (Goßmann et al. 2024) and insect activity (Lindman et al. 2023, Tielens and Kelly 2024) in the south compared to the north. In contrast, brown-rot fungi may be more closely adapted to vectoring beetles resulting in positive relationships both in the south and in the north.

Only brown-rot fungi showed a clear response to microclimate. In warmer regions, richness of brown rot fungi was unaffected by shade level, suggesting that even shaded logs offered suitable conditions. In contrast, in the north, richness increased markedly with sun exposure—implying that microclimate becomes a limiting factor under cooler regional climates. This difference in habitat requirements between regions is consistent with the “hydroclimatic compensation model” (Ackerly et al. 2020). Conclusively, our findings underscore the importance of considering functional group–specific responses when predicting fungal diversity patterns across environmental gradients.

## Conclusion

We did not find support for a latitudinal diversity gradient for all wood-inhabiting fungi. Furthermore, our results showed that different functional groups respond differently to environmental drivers. The diversity of brown-rot fungi followed the hydroclimatic compensation model: there was a marked increase in species richness with sun exposure in colder regions, whereas no such pattern was observed in warmer regions. Moreover, brown-rot richness was positively correlated to beetle abundance, suggesting that beetle-facilitated dispersal and favorable microclimate may jointly reinforce brown-rot diversity in sun-exposed habitats. In contrast, white-rot fungi and the broader fungal community did not show clear responses to either factor, indicating that there is no uniformly applicable diversity pattern across fungal functional groups.

The choice of forestry systems affects the canopy cover, and thus, affects microclimatic temperatures. Moreover, beetles acting as vectors, including forest pests, can increase with a warming climate, and various changes in management practices (Lindman et al. 2023). Given our results, a warming climate and changes in forestry can influence especially brown-rot fungi and that can lead to shifts in the overall fungal species composition. Due to the contrasting decomposition efficiency of brown- and white-rotters (Highley and Kirk 1979), shifts in species composition can affect dead wood decomposition, which is an essential ecological function in forest ecosystems.

## Supplementary Material

fiaf116_Supplemental_File

## Data Availability

Accession number: PRJNA1225487 in SRA.
