# Supplementary material for: Contrasting diversity patterns of brown: and white-rot wood saprotrophs in response to climate and dispersal vectors
Source: FEMS Microbiol Ecol. 2025 Nov 21;102(1):fiaf116. doi: 10.1093/femsec/fiaf116 (PMC12724080; doi:10.1093/femsec/fiaf116)
Supplement: fiaf116_Supplemental_File [file fiaf116_supplemental_file.docx]

**Appendix S1**

Table S1: Wald Chi-square test of generalized linear mixed model using the combined dataset, and dataset 1 and 2 separately. Dataset 1 refers to the data sampled along the whole log and pooled on plot shade level prior to sequencing, whereas dataset 2 refers to data sampled per wood disc, which were not pooled, but aggregated on plot shade level during analysis. Richness of all wood-inhabiting fungi, and brown- and white-rotters were used as response variables. Study plot nested in study region was used as random effect in all models irrespective of the dataset. Numbers in bold indicate a significant effect (<0.05), numbers in italic indicate a marginal significant effect (<0.1). The marginal R^2^ refers to the measured proportion of variance explained only by the fixed effects, whereas the conditional R^2^ accounts for both fixed and random effects.

|  | **Combined dataset** | | | | **Dataset 1** | | | | **Dataset 2** | | | |
| --- | --- | --- | --- | --- | --- | --- | --- | --- | --- | --- | --- | --- |
| **Model with predictor variables** | **Marginal and conditional R*^2^*** | ***χ*^2^** | **DF** | ***p*-value** | **Marginal and conditional R*^2^*** | ***χ*^2^** | **DF** | ***p*-value** | **Marginal and conditional R*^2^*** | ***χ*^2^** | **DF** | ***p*-value** |
| **All fungi** | 0.16; 0.41 |  |  |  | 0.14; 0.29 |  |  |  | 0.12; 0.38 |  |  |  |
| Shade level |  | 2.73 | 2 | 0.26 |  | 0.75 | 2 | 0.69 |  | 2.93 | 2 | 0.23 |
| Regional climate |  | 2.69 | 1 | 0.1 |  | 1.47 | 1 | 0.23 |  | 1.57 | 1 | 0.21 |
| Beetle abundance |  | 2.13 | 1 | 0.14 |  | 4.11 | 1 | **0.01** |  | 0.75 | 1 | 0.39 |
| Regional climate: Shade level |  | 0.46 | 2 | 0.79 |  | 0.88 | 2 | 0.64 |  | 0.56 | 2 | 0.75 |
|  |  |  |  |  |  |  |  |  |  |  |  |  |
| **Brown-rotters** | 0.23; 0.39 |  |  |  | 0.41; 0.53 |  |  |  | 0.17; 0.4 |  |  |  |
| Shade level |  | 4.48 | 2 | 0.11 |  | 13.0 | 2 | **0.001** |  | 1.91 | 2 | 0.39 |
| Regional climate |  | 1.71 | 1 | 0.19 |  | 0.02 | 1 | 0.9 |  | 2.6 | 1 | 0.11 |
| Beetle abundance |  | 10.5 | 1 | **0.001** |  | 2.27 | 1 | 0.13 |  | 8.53 | 1 | **0.003** |
| Regional climate: Shade level |  | 4.71 | 2 | *0.09* |  | 12.4 | 2 | **0.002** |  | 1.84 | 2 | 0.39 |
|  |  |  |  |  |  |  |  |  |  |  |  |  |
| **White-rotters** | 0.06; 0.55 |  |  |  | 0.09; 0.34 |  |  |  | 0.05; 0.42 |  |  |  |
| Shade level |  | 0.1 | 2 | 0.95 |  | 0.35 | 2 | 0.84 |  | 1.49 | 2 | 0.47 |
| Regional climate |  | 0.67 | 1 | 0.41 |  | 3.32 | 1 | *0.07* |  | 1.19 | 1 | 0.27 |
| Beetle abundance |  | 1.05 | 1 | 0.31 |  | 0.02 | 1 | 0.89 |  | 0.18 | 1 | 0.67 |
| Regional climate: Shade level |  | 2.43 | 2 | 0.29 |  | 2.09 | 2 | 0.35 |  | 2.64 | 2 | 0.27 |

Table S2: Summary table of the generalized linear mixed models using the combined dataset, and dataset 1 and 2 separately. Dataset 1 refers to the data sampled along the whole log and pooled on plot shade level prior to sequencing, whereas dataset 2 refers to data sampled per wood disc, which were not pooled, but aggregated on plot shade level during the analysis. Richness of all wood-inhabiting fungi, and brown- and white-rotters was used as response variables. Shade level, regional temperature, the interaction between regional temperature and shade level, and log-transformed beetle abundance were used as explanatory variables. Study plot nested in study region was used as random effect in all models irrespective of the dataset. Shade II refers to intermediately shaded, and shade III to fully shaded. Estimate and Std. error were extracted from the summary table, whereas degrees of freedom were calculated with the function *df.residual* and the lower and upper confidence intervals were calculated with the function *confint*. The respective *p*-value can be found in table S1.

| **Combined dataset** | | | | | | **Dataset 1** | | | | | **Dataset 2** | | | | | |
| --- | --- | --- | --- | --- | --- | --- | --- | --- | --- | --- | --- | --- | --- | --- | --- | --- |
| **Model with predictor variables** | **Estimate** | **Std. Error** | **DF** | **Lower CI** | **Upper CI** | **Estimate** | **Std. Error** | **DF** | **Lower CI** | **Upper CI** | **Estimate** | **Std. Error** | **DF** | **Lower CI** | **Upper CI** |  |
| **All fungi** |  |  |  |  |  |  |  |  |  |  |  |  |  |  |  |  |
| Shade level |  |  |  |  |  |  |  |  |  |  |  |  |  |  |  |  |
| Shade II | -0.38 | 0.63 | 71 | -1.61 | 0.85 | -0.79 | 0.89 | 71 | -2.52 | 0.95 | -0.43 | 0.68 | 71 | -1.77 | 0.91 |  |
| Shade III | -0.49 | 0.62 | 71 | -1.71 | 0.71 | -0.12 | 0.89 | 71 | -1.87 | 1.63 | -0.59 | 0.67 | 71 | -1.91 | 0.72 |  |
| Beetle abundance | 0.05 | 0.04 | 71 | -0.02 | 0.12 | 0.09 | 0.05 | 71 | 0.003 | 0.19 | 0.03 | 0.04 | 71 | -0.04 | 0.12 |  |
| Regional temperature | 0.03 | 0.05 | 71 | -0.06 | 0.12 | 0.02 | 0.06 | 71 | -0.09 | 0.14 | 0.03 | 0.05 | 71 | -0.08 | 0.13 |  |
| Regional temperature: Shade level |  |  |  |  |  |  |  |  |  |  |  |  |  |  |  |  |
| Regional temperature: Shade II | 0.03 | 0.05 | 71 | -0.07 | 0.13 | 0.06 | 0.07 | 71 | -0.08 | 0.19 | 0.03 | 0.06 | 71 | -0.07 | 0.14 |  |
| Regional temperature: Shade III | 0.03 | 0.05 | 71 | -0.07 | 0.13 | 0.002 | 0.07 | 71 | -0.14 | 0.14 | 0.04 | 0.05 | 71 | -0.07 | 0.14 |  |
|  |  |  |  |  |  |  |  |  |  |  |  |  |  |  |  |  |
| **Brown-rotters** |  |  |  |  |  |  |  |  |  |  |  |  |  |  |  |  |
| Shade level |  |  |  |  |  |  |  |  |  |  |  |  |  |  |  |  |
| Shade II | -2.87 | 1.34 | 72 | -5.49 | -0.25 | -7.15 | 2.21 | 72 | -11.5 | -2.82 | -1.43 | 1.44 | 72 | -4.24 | 1.39 |  |
| Shade III | -2.63 | 1.41 | 72 | -5.38 | 0.13 | -9.4 | 3.42 | 72 | -16.1 | -2.71 | -2.19 | 1.49 | 72 | -5.12 | 0.72 |  |
| Beetle abundance | 0.26 | 0.08 | 72 | 0.1 | 0.41 | 0.18 | 0.12 | 72 | -0.05 | 0.42 | 0.25 | 0.09 | 72 | 0.08 | 0.42 |  |
| Regional temperature | -0.22 | 0.09 | 72 | -0.4 | -0.04 | -0.27 | 0.13 | 72 | -0.53 | -0.01 | -0.21 | 0.1 | 72 | -0.41 | -0.01 |  |
| Regional temperature: Shade level |  |  |  |  |  |  |  |  |  |  |  |  |  |  |  |  |
| Regional temperature: Shade II | 0.22 | 0.11 | 72 | 0.004 | 0.43 | 0.53 | 0.18 | 72 | 0.19 | 0.89 | 0.1 | 0.12 | 72 | -0.13 | 0.34 |  |
| Regional temperature: Shade III | 0.19 | 0.11 | 72 | -0.04 | 0.41 | 0.64 | 0.26 | 72 | 0.13 | 1.15 | 0.16 | 0.12 | 72 | -0.08 | 0.39 |  |
|  |  |  |  |  |  |  |  |  |  |  |  |  |  |  |  |  |
| **White-rotters** |  |  |  |  |  |  |  |  |  |  |  |  |  |  |  |  |
| Shade level |  |  |  |  |  |  |  |  |  |  |  |  |  |  |  |  |
| Shade II | 1.78 | 4.67 | 71 | -7.39 | 10.9 | 5.57 | 4.16 | 71 | -2.59 | 13.7 | -5.38 | 4.87 | 71 | -14.93 | 4.17 |  |
| Shade III | -5.08 | 4.61 | 71 | -14.1 | 3.95 | 1.88 | 4.12 | 71 | -6.19 | 9.95 | -6.66 | 4.81 | 71 | -16.02 | 2.78 |  |
| Beetle abundance | -0.31 | 0.3 | 71 | -0.9 | 0.28 | -0.03 | 0.24 | 71 | -0.5 | 0.44 | 0.12 | 0.29 | 71 | -0.45 | 0.05 |  |
| Regional temperature | -0.37 | 0.42 | 71 | -1.19 | 0.45 | -0.16 | 0.29 | 71 | -0.75 | 0.42 | -0.69 | 0.38 | 71 | -1.43 | 0.69 |  |
| Regional temperature: Shade level |  |  |  |  |  |  |  |  |  |  |  |  |  |  |  |  |
| Regional temperature: Shade II | -0.13 | 0.38 | 71 | -0.88 | 0.61 | -0.48 | 0.34 | 71 | -1.14 | 0.18 | 0.46 | 0.39 | 71 | -0.32 | 1.23 |  |
| Regional temperature: Shade III | 0.43 | 0.37 | 71 | -0.31 | 1.16 | -0.16 | 0.33 | 71 | -0.82 | 0.49 | 0.61 | 0.39 | 71 | -0.15 | 1.38 |  |

Table S3: Wald Chi-square test of generalized linear mixed model using the combined dataset, and dataset 1 and 2 separately. Dataset 1 refers to the data sampled along the whole log and pooled on plot shade level prior to sequencing, whereas dataset 2 refers to data sampled per wood disc, which were not pooled, but aggregated on plot shade level during the analysis. Relative abundance of brown- and white-rotters were used as response variables. Study plot nested in study region was used as random effect in all models irrespective of the dataset. Numbers in bold indicate a significant effect (<0.05), numbers in italic indicate a marginal significant effect (<0.1). The marginal R^2^ refers to the measured proportion of variance explained only by the fixed effects, whereas the conditional R^2^ accounts for both fixed and random effects.

|  | **Combined dataset** | | | | **Dataset 1** | | | | **Dataset 2** | | | |
| --- | --- | --- | --- | --- | --- | --- | --- | --- | --- | --- | --- | --- |
| **Model with predictor variables** | **Marginal and conditional R*^2^*** | ***χ*^2^** | **DF** | ***p*-value** | **Marginal and conditional R*^2^*** | ***χ*^2^** | **DF** | ***p*-value** | **Marginal and conditional R*^2^*** | ***χ*^2^** | **DF** | ***p*-value** |
|  |  |  |  |  |  |  |  |  |  |  |  |  |
| **Brown-rotters** | 1; 1 |  |  |  | 1; 1 |  |  |  | 0.22; 1 |  |  |  |
| Shade level |  | 14.2 | 2 | **<0.001** |  | 23.4 | 2 | **<0.001** |  | 4.2 | 2 | 0.12 |
| Regional climate |  | 1.27 | 1 | 0.26 |  | 0.01 | 1 | 0.94 |  | 1.53 | 1 | 0.22 |
| Beetle abundance |  | 4.39 | 1 | **0.04** |  | 3.26 | 1 | *0.07* |  | 4.02 | 1 | **0.05** |
| Regional climate: Shade level |  | 1.27 | 2 | 0.53 |  | 1.56 | 2 | 0.46 |  | 0.54 | 2 | 0.77 |
|  |  |  |  |  |  |  |  |  |  |  |  |  |
| **White-rotters** | 0.06; 0.06 |  |  |  | 0.04; 0.04 |  |  |  | 0.12; 0.17 |  |  |  |
| Shade level |  | 0.59 | 2 | 0.74 |  | 1.2 | 2 | 0.55 |  | 0.05 | 2 | 0.97 |
| Regional climate |  | 0.56 | 1 | 0.46 |  | 0.56 | 1 | 0.46 |  | 1.29 | 1 | 0.26 |
| Beetle abundance |  | 0.44 | 1 | 0.51 |  | 0.002 | 1 | 0.96 |  | 1.38 | 1 | 0.24 |
| Regional climate: Shade level |  | 0.48 | 2 | 0.79 |  | 0.07 | 2 | 0.97 |  | 0.26 | 2 | 0.88 |

Table S4: Summary table of the generalized linear mixed models using the combined dataset, and dataset 1 and 2 separately. Dataset 1 refers to the data sampled along the whole log and pooled on plot shade level prior to sequencing, whereas dataset 2 refers to data sampled per wood disc, which were not pooled, but aggregated on plot shade level during the analysis. Relative abundance of brown- and white-rotters was used as response variables. Shade level, regional temperature, the interaction between regional temperature and shade level, and log-transformed beetle abundance were used as explanatory variables. Study plot nested in study region was used as random effect in all models irrespective of the dataset. Shade II refers to intermediately shaded, and shade III to fully shaded. Estimate and Std. error were extracted from the summary table, whereas degrees of freedom were calculated with the function *df.residual* and the lower and upper confidence intervals were calculated with the function *confint*. The respective *p*-value can be found in table S3.

| **Combined dataset** | | | | | | **Dataset 1** | | | | | **Dataset 2** | | | | | |
| --- | --- | --- | --- | --- | --- | --- | --- | --- | --- | --- | --- | --- | --- | --- | --- | --- |
| **Model with predictor variables** | **Estimate** | **Std. Error** | **DF** | **Lower CI** | **Upper CI** | **Estimate** | **Std. Error** | **DF** | **Lower CI** | **Upper CI** | **Estimate** | **Std. Error** | **DF** | **Lower CI** | **Upper CI** |  |
|  |  |  |  |  |  |  |  |  |  |  |  |  |  |  |  |  |
| **Brown-rotters** |  |  |  |  |  |  |  |  |  |  |  |  |  |  |  |  |
| Shade level |  |  |  |  |  |  |  |  |  |  |  |  |  |  |  |  |
| Shade II | -2.61 | 3.37 | 70 | -9.22 | 4.0 | -2.99 | 4.03 | 71 | -10.9 | 4.91 | -3.09 | 3.25 | 70 | -9.46 | 3.28 |  |
| Shade III | 1.18 | 2.93 | 70 | -4.55 | 6.92 | -10.9 | 6.73 | 71 | -24.1 | 2.34 | -1.76 | 2.98 | 70 | -7.59 | 4.08 |  |
| Beetle abundance | 0.31 | 0.15 | 70 | 0.02 | 0.6 | 0.32 | 0.18 | 71 | -0.03 | 0.66 | 0.37 | 0.19 | 70 | 0.01 | 0.74 |  |
| Regional temperature | -0.09 | 0.17 | 70 | -0.43 | 0.24 | -0.07 | 0.18 | 71 | -0.43 | 0.29 | -0.32 | 0.23 | 70 | -0.78 | 0.14 |  |
| Regional temperature: Shade level |  |  |  |  |  |  |  |  |  |  |  |  |  |  |  |  |
| Regional temperature: Shade II | 0.11 | 0.27 | 70 | -0.42 | 0.65 | 0.12 | 0.32 | 71 | -0.51 | 0.75 | 0.19 | 0.27 | 70 | -0.33 | 0.2 |  |
| Regional temperature: Shade III | -0.19 | 0.24 | 70 | -0.67 | 0.27 | 0.64 | 0.52 | 71 | -0.38 | 1.66 | 0.11 | 0.25 | 70 | -0.37 | 0.59 |  |
|  |  |  |  |  |  |  |  |  |  |  |  |  |  |  |  |  |
| **White-rotters** |  |  |  |  |  |  |  |  |  |  |  |  |  |  |  |  |
| Shade level |  |  |  |  |  |  |  |  |  |  |  |  |  |  |  |  |
| Shade II | -0.7 | 1.58 | 72 | -3.79 | 2.39 | 0.76 | 1.94 | 71 | -3.03 | 4.56 | -0.84 | 1.76 | 72 | -4.28 | 2.6 |  |
| Shade III | 0.21 | 1.53 | 72 | -2.78 | 3.21 | 0.56 | 1.94 | 71 | -3.24 | 4.36 | -0.35 | 1.71 | 72 | -3.69 | 2.99 |  |
| Beetle abundance | 0.05 | 0.08 | 72 | -0.09 | 0.2 | -0.004 | 0.09 | 71 | -0.18 | 0.17 | 0.1 | 0.09 | 72 | -0.07 | 0.28 |  |
| Regional temperature | 0.02 | 0.09 | 72 | -0.16 | 0.2 | 0.08 | 0.12 | 71 | -0.16 | 0.32 | 0.05 | 0.12 | 72 | -0.18 | 0.28 |  |
| Regional temperature: Shade level |  |  |  |  |  |  |  |  |  |  |  |  |  |  |  |  |
| Regional temperature: Shade II | 0.07 | 0.13 | 72 | -0.18 | 0.32 | -0.04 | 0.16 | 71 | -0.35 | 0.27 | 0.07 | 0.14 | 72 | -0.21 | 0.35 |  |
| Regional temperature: Shade III | -0.01 | 0.12 | 72 | -0.25 | 0.23 | -0.03 | 0.16 | 71 | -0.33 | 0.28 | 0.03 | 0.14 | 72 | -0.24 | 0.29 |  |

Table S5: Summary table of the PERMANOVA result using the combined dataset, and dataset 1 and 2 separately. Dataset 1 refers to the data sampled along the whole log and pooled on plot shade level prior to sequencing, whereas dataset 2 refers to data sampled per wood disc, which were not pooled, but aggregated on plot shade level during the analysis. Community composition of all wood-inhabiting fungi and brown- and white-rotters was used as response variables, and regional temperature, shade level, and log-transformed beetle abundance as explanatory variables.

|  |  | **Combined dataset** | | | **Dataset 1** | | | **Dataset 2** | | |
| --- | --- | --- | --- | --- | --- | --- | --- | --- | --- | --- |
|  | **Predictor variable** | **R^2^** | **F-value** | **p-value** | **R^2^** | **F-value** | **p-value** | **R^2^** | **F-value** | **p-value** |
| **All fungi** |  |  |  |  |  |  |  |  |  |  |
|  | Shade level | 0.03 | 1.3 | **0.001** | 0.03 | 1.02 | 0.22 | 0.02 | 0.99 | *0.06* |
|  | Regional temperature | 0.07 | 5.74 | *0.06* | 0.01 | 1.18 | 0.12 | 0.07 | 5.67 | **0.05** |
|  | Beetle abundance | 0.02 | 1.3 | 0.33 | 0.01 | 0.86 | 0.38 | 0.02 | 1.49 | 0.57 |
|  |  |  |  |  |  |  |  |  |  |  |
| **Brown-rotters** |  |  |  |  |  |  |  |  |  |  |
|  | Shade level | 0.07 | 2.22 | **0.003** | 0.06 | 0.91 | 0.54 | 0.04 | 1.14 | 0.13 |
|  | Regional temperature | 0.05 | 3.65 | 0.14 | 0.01 | 0.44 | 0.53 | 0.05 | 3.16 | 0.69 |
|  | Beetle abundance | 0.01 | 0.92 | 0.31 | 0.02 | 0.72 | 0.71 | 0.01 | 0.33 | 0.95 |
|  |  |  |  |  |  |  |  |  |  |  |
| **White-rotters** |  |  |  |  |  |  |  |  |  |  |
|  |  |  |  |  |  |  |  |  |  |  |
|  | Shade level | 0.02 | 0.95 | 0.38 | 0.03 | 1.03 | 0.33 | 0.02 | 0.78 | 0.65 |
|  | Regional temperature | 0.14 | 13.5 | 0.19 | 0.02 | 1.19 | 0.74 | 0.12 | 10.8 | 0.24 |
|  | Beetle abundance | 0.02 | 1.81 | 0.11 | 0.02 | 1.23 | 0.63 | 0.02 | 2.02 | 0.29 |

Table S6: Summary table of the generalized linear mixed models with richness of all wood-inhabiting fungi, and brown- and white-rotters as response variables separated into south and north. Shade level and log-transformed beetle abundance were included as explanatory variables. Shade II refers to intermediately shaded, and shade III to fully shaded. Estimate and Std. error were extracted from the summary table, whereas degrees of freedom were calculated with the function *df.residual* and the lower and upper confidence intervals were calculated with the function *confint*. The respective *p*-value can be found in table 3 in the main text.

| **Predictor variables** | **Estimate** | **Std. Error** | **DF** | **Lower CI** | **Upper CI** |
| --- | --- | --- | --- | --- | --- |
| **South** |  |  |  |  |  |
| **All fungi** |  |  |  |  |  |
| Shade level |  |  |  |  |  |
| Shade level II | 0.09 | 0.11 | 32 | -0.13 | 0.3 |
| Shade level III | 0.02 | 0.12 | 32 | -0.2 | 0.25 |
| Beetle abundance | 0.18 | 0.05 | 32 | 0.08 | 0.28 |
|  |  |  |  |  |  |
| **Brown-rotters** |  |  |  |  |  |
| Shade level |  |  |  |  |  |
| Shade level II | 0.26 | 0.29 | 33 | -0.32 | 0.84 |
| Shade level III | 0.16 | 0.33 | 33 | -0.48 | 0.81 |
| Beetle abundance | 0.53 | 0.19 | 33 | 0.16 | 0.89 |
|  |  |  |  |  |  |
| **White-rotters** |  |  |  |  |  |
| Shade level |  |  |  |  |  |
| Shade level II | 0.22 | 0.78 | 32 | -1.3 | 1.75 |
| Shade level III | 1.09 | 0.83 | 32 | -0.53 | 2.73 |
| Beetle abundance | -0.16 | 0.49 | 32 | -1.12 | 0.79 |
|  |  |  |  |  |  |
| **North** |  |  |  |  |  |
|  |  |  |  |  |  |
| **All fungi** |  |  |  |  |  |
| Shade level |  |  |  |  |  |
| Shade level II | 0.01 | 0.11 | 35 | -0.21 | 0.22 |
| Shade level III | -0.21 | 0.11 | 35 | -0.42 | 0.01 |
| Beetle abundance | -0.02 | 0.05 | 35 | -0.11 | 0.07 |
|  |  |  |  |  |  |
| **Brown-rotters** |  |  |  |  |  |
| Shade level |  |  |  |  |  |
| Shade level II | -1.09 | 0.35 | 35 | -1.78 | -0.4 |
| Shade level III | -1.45 | 0.36 | 35 | -2.15 | -0.75 |
| Beetle abundance | 0.4 | 0.13 | 35 | 0.16 | 0.65 |
|  |  |  |  |  |  |
| **White-rotters** |  |  |  |  |  |
| Shade level |  |  |  |  |  |
| Shade level II | 0.02 | 0.12 | 36 | -0.22 | 0.25 |
| Shade level III | -0.06 | 0.12 | 36 | -0.3 | 0.18 |
| Beetle abundance | -0.03 | 0.04 | 36 | -0.11 | 0.06 |

Table S7: Wald Chi-square test of generalized linear mixed model using the combined dataset, and dataset 1 and 2 separately. The datasets were separated into south (Tönnersjöheden, Asa, Siljansfors) and north (Vindeln, Järpen, Ätnarova). Dataset 1 refers to the data sampled along the whole log and pooled on plot shade level prior to sequencing, whereas dataset 2 refers to data sampled per wood disc, which were not pooled, but aggregated on plot shade level during the analysis. Richness of all wood-inhabiting fungi, and brown- and white-rotters were used as response variables. Study plot nested in study region was used as random effect in all models irrespective of the dataset. Numbers in bold indicate a significant effect (<0.05), numbers in italic indicate a marginal significant effect (<0.1). The marginal R^2^ refers to the measured proportion of variance explained only by the fixed effects, whereas the conditional R^2^ accounts for both fixed and random effects.

|  | **Combined dataset** | | | | **Dataset 1** | | | | **Dataset 2** | | | |  |
| --- | --- | --- | --- | --- | --- | --- | --- | --- | --- | --- | --- | --- | --- |
| **SOUTH** |  | | | |  | | | |  | | | |  |
| **Model with predictor variables** | **Marginal and conditional R*^2^*** | ***χ*^2^** | **DF** | ***p*-value** | **Marginal and conditional R*^2^*** | ***χ*^2^** | **DF** | ***p*-value** | **Marginal and conditional R*^2^*** | ***χ*^2^** | **DF** | ***p*-value** |  |
| **All fungi** | 0.31; 0.42 |  |  |  | 0.08; 0.18 |  |  |  | 0.22; 0.32 |  |  |  |  |
| Shade level |  | 0.69 | 2 | 0.71 |  | 0.69 | 2 | 0.71 |  | 0.54 | 2 | 0.76 |  |
| Beetle abundance |  | 11.9 | 1 | **<0.001** |  | 1.94 | 1 | 0.16 |  | 1.84 | 1 | 0.17 |  |
|  |  |  |  |  |  |  |  |  |  |  |  |  |  |
| **Brown-rotters** | 0.29; 0.63 |  |  |  | 0.36; 0.56 |  |  |  | 0.17; 0.4 |  |  |  |  |
| Shade level |  | 0.79 | 2 | 0.67 |  | 2.32 | 2 | 0.31 |  | 0.29 | 2 | 0.86 |  |
| Beetle abundance |  | 7.86 | 1 | **0.005** |  | 8.24 | 1 | **0.004** |  | 6.39 | 1 | **0.01** |  |
|  |  |  |  |  |  |  |  |  |  |  |  |  |  |
| **White-rotters** | 0.04; 0.51 |  |  |  | 0.01; 0.24 |  |  |  | 0.21; 0.29 |  |  |  |  |
| Shade level |  | 2.08 | 2 | 0.36 |  | 0.1 | 2 | 0.95 |  | 5.39 | 2 | **0.02** |  |
| Beetle abundance |  | 0.11 | 1 | 0.74 |  | 0.09 | 1 | 0.76 |  | 7.05 | 1 | **0.03** |  |
| **NORTH** |  |  |  |  |  |  |  |  |  |  |  |  |  |
| **All fungi** | | 0.05; 0.55 |  |  |  | 0.09; 0.31 |  |  |  | 0.07; 0.55 |  |  |  |
| Shade level | |  | 4.38 | 2 | 0.11 |  | 1.44 | 2 | 0.49 |  | 4.97 | 2 | *0.08* |
| Beetle abundance | |  | 0.24 | 1 | 0.62 |  | 2.72 | 1 | *0.09* |  | 0.91 | 1 | 0.34 |
|  | |  |  |  |  |  |  |  |  |  |  |  |  |
| **Brown-rotters** | | 0.39; 0.58 |  |  |  | 0.46; 0.53 |  |  |  | 0.2; 0.43 |  |  |  |
| Shade level | |  | 18.6 | 2 | **<0.001** |  | 37.9 | 2 | **<0.001** |  | 6.99 | 2 | **0.03** |
| Beetle abundance | |  | 10.3 | 1 | **0.001** |  | 0.33 | 1 | 0.57 |  | 3.0 | 1 | *0.08* |
|  | |  |  |  |  |  |  |  |  |  |  |  |  |
| **White-rotters** | | 0.01; 0.37 |  |  |  | 0.01; 0.36 |  |  |  | 0.002; 0.34 |  |  |  |
| Shade level | |  | 0.41 | 2 | 0.82 |  | 0.94 | 2 | 0.63 |  | 0.1 | 2 | 0.94 |
| Beetle abundance | |  | 0.38 | 1 | 0.54 |  | 0.01 | 1 | 0.92 |  | 0.002 | 1 | 0.97 |

Table S8: Summary table of the generalized linear mixed models using the combined dataset, and dataset 1 and 2 separately, separated into south and north. Dataset 1 refers to the data sampled along the whole log and pooled on plot shade level prior to sequencing, whereas dataset 2 refers to data sampled per wood disc, which were not pooled, but aggregated on plot shade level during the analysis. Study plot nested in study region was used as random effect in all models irrespective of the dataset. Shade level and log-transformed beetle abundance were included as explanatory variables. Shade II refers to intermediately shaded, and shade III to fully shaded. Estimate and Std. error were extracted from the summary table, whereas degrees of freedom were calculated with the function *df.residual* and the lower and upper confidence intervals were calculated with the function *confint*. The respective *p*-value can be found in table S7.

| **Combined dataset** | | | | | | **Dataset 1** | | | | | **Dataset 2** | | | | | |
| --- | --- | --- | --- | --- | --- | --- | --- | --- | --- | --- | --- | --- | --- | --- | --- | --- |
| **SOUTH** | | | | | |  | | | | |  | | | | | |
| **Model with predictor variables** | **Estimate** | **Std. Error** | **DF** | **Lower CI** | **Upper CI** | **Estimate** | **Std. Error** | **DF** | **Lower CI** | **Upper CI** | **Estimate** | **Std. Error** | **DF** | **Lower CI** | **Upper CI** |  |
| **All fungi** |  |  |  |  |  |  |  |  |  |  |  |  |  |  |  |  |
| Shade level |  |  |  |  |  |  |  |  |  |  |  |  |  |  |  |  |
| Shade II | 0.09 | 0.11 | 32 | -0.13 | 0.3 | 0.07 | 0.18 | 32 | -0.27 | 0.42 | 0.09 | 0.14 | 32 | -0.19 | 0.36 |  |
| Shade III | 0.02 | 0.12 | 32 | -0.2 | 0.25 | -0.07 | 0.18 | 32 | -0.43 | 0.29 | 0.02 | 0.16 | 32 | -0.29 | 0.33 |  |
| Beetle abundance | 0.18 | 0.05 | 32 | 0.08 | 0.28 | 0.1 | 0.07 | 32 | -0.04 | 0.25 | 0.16 | 0.12 | 32 | -0.07 | 0.39 |  |
|  |  |  |  |  |  |  |  |  |  |  |  |  |  |  |  |  |
| **Brown-rotters** |  |  |  |  |  |  |  |  |  |  |  |  |  |  |  |  |
| Shade level |  |  |  |  |  |  |  |  |  |  |  |  |  |  |  |  |
| Shade II | 0.26 | 0.29 | 33 | -0.32 | 0.84 | 0.59 | 0..45 | 33 | -0.28 | 1.47 | 0.05 | 0.34 | 33 | -0.62 | 0.71 |  |
| Shade III | 0.16 | 0.33 | 33 | -0.48 | 0.81 | 0.19 | 0.55 | 33 | -0.89 | 1.26 | 0.19 | 0.37 | 33 | -0.54 | 0.92 |  |
| Beetle abundance | 0.53 | 0.19 | 33 | 0.16 | 0.89 | 0.77 | 0.27 | 33 | 0.24 | 1.29 | 0.53 | 0.21 | 33 | 0.12 | 0.94 |  |
|  |  |  |  |  |  |  |  |  |  |  |  |  |  |  |  |  |
| **White-rotters** |  |  |  |  |  |  |  |  |  |  |  |  |  |  |  |  |
| Shade level |  |  |  |  |  |  |  |  |  |  |  |  |  |  |  |  |
| Shade II | 0.22 | 0.78 | 32 | -1.3 | 1.75 | -0.21 | 0.68 | 32 | -1.55 | 1.12 | 0.68 | 0.83 | 32 | -0.96 | 2.31 |  |
| Shade III | 1.09 | 0.83 | 32 | -0.53 | 2.73 | -0.09 | 0.72 | 32 | -1.49 | 1.31 | 2.21 | 0.86 | 32 | 0.53 | 3.9 |  |
| Beetle abundance | -0.16 | 0.49 | 32 | -1.12 | 0.79 | -0.11 | 0.36 | 32 | -0.83 | 0.6 | 0.84 | 0.36 | 32 | 0.13 | 1.54 |  |
| **NORTH** |  |  |  |  |  |  |  |  |  |  |  |  |  |  |  |  |
| **All fungi** |  |  |  |  |  |  |  |  |  |  |  |  |  |  |  |  |
| Shade level |  |  |  |  |  |  |  |  |  |  |  |  |  |  |  |  |
| Shade II | 0.01 | 0.11 | 35 | -0.21 | 0.22 | -0.19 | 0.16 | 35 | -0.51 | 0.13 | 0.01 | 0.12 | 35 | -0.22 | 0.24 |  |
| Shade III | -0.21 | 0.11 | 35 | -0.42 | 0.01 | -0.12 | 0.16 | 35 | -0.44 | 0.2 | -0.24 | 0.12 | 35 | -0.47 | -0.01 |  |
| Beetle abundance | -0.02 | 0.05 | 35 | -0.11 | 0.07 | 0.09 | 0.06 | 35 | -0.02 | 0.21 | -0.05 | 0.05 | 35 | -0.14 | 0.05 |  |
|  |  |  |  |  |  |  |  |  |  |  |  |  |  |  |  |  |
| **Brown-rotters** |  |  |  |  |  |  |  |  |  |  |  |  |  |  |  |  |
| Shade level |  |  |  |  |  |  |  |  |  |  |  |  |  |  |  |  |
| Shade II | -1.09 | 0.35 | 35 | -1.78 | -0.4 | -1.53 | 0.34 | 35 | -2.18 | -0.87 | -0.54 | 0.44 | 35 | -1.39 | 0.32 |  |
| Shade III | -1.45 | 0.36 | 35 | -2.15 | -0.75 | -1.96 | 0.34 | 35 | -2.63 | -1.29 | -1.18 | 0.45 | 35 | -2.06 | -0.31 |  |
| Beetle abundance | 0.4 | 0.13 | 35 | 0.16 | 0.65 | 0.06 | 0.11 | 35 | -0.15 | 0.28 | 0.27 | 0.16 | 35 | -0.04 | 0.58 |  |
|  |  |  |  |  |  |  |  |  |  |  |  |  |  |  |  |  |
| **White-rotters** |  |  |  |  |  |  |  |  |  |  |  |  |  |  |  |  |
| Shade level |  |  |  |  |  |  |  |  |  |  |  |  |  |  |  |  |
| Shade II | 0.02 | 0.12 | 36 | -0.22 | 0.25 | -0.18 | 0.21 | 36 | -0.59 | 0.24 | 0.05 | 0.16 | 36 | -0.26 | 0.36 |  |
| Shade III | -0.06 | 0.12 | 36 | -0.3 | 0.18 | -0.17 | 0.22 | 36 | -0.59 | 0.25 | 0.03 | 0.16 | 36 | -0.28 | 0.35 |  |
| Beetle abundance | -0.03 | 0.04 | 36 | -0.11 | 0.06 | 0.007 | 0.07 | 36 | -0.14 | 0.15 | -0.002 | 0.06 | 36 | -0.11 | 0.11 |  |


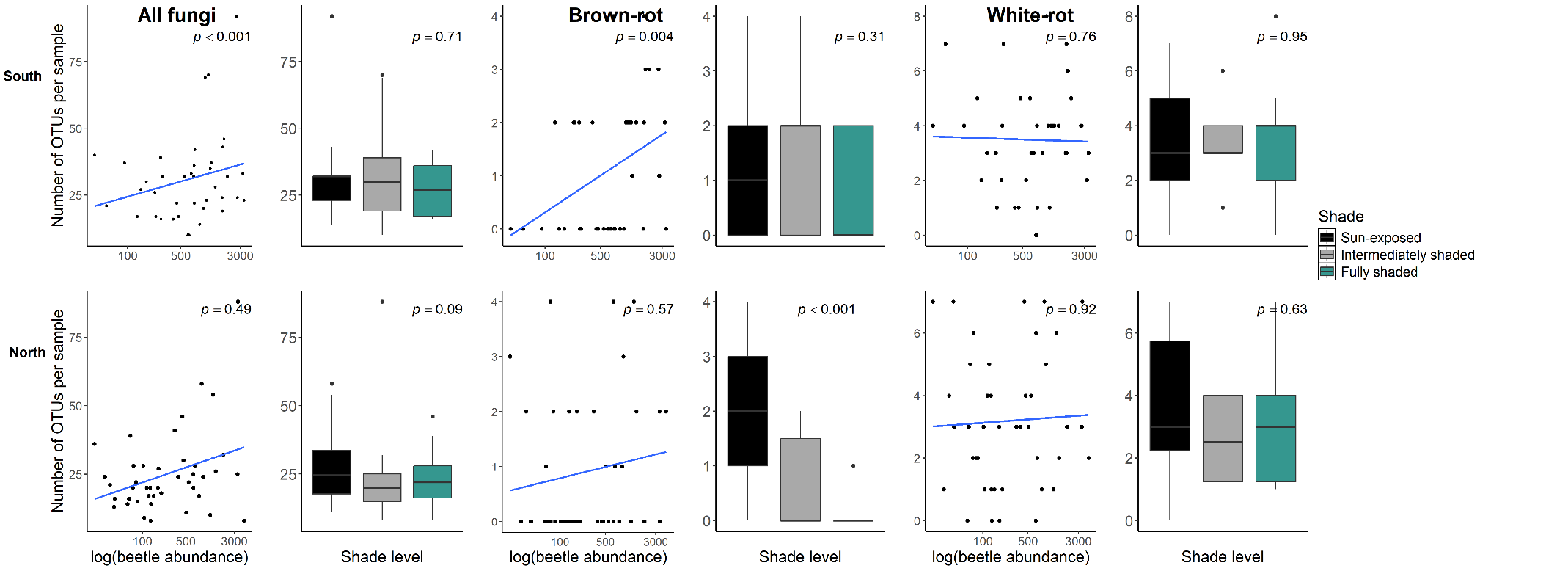


Figure S1. Dataset 1: Species richness in spruce logs of all fungi, brown-rotters, and white-rotters along a beetle abundance gradient (log-transformed), and along a shade gradient (respective boxplots to the right). The upper panels show the effects in the south (Tönnersjöheden, Asa, Siljansfors), and the lower panels the effects in the north (Vindeln, Järpen, Ätnarova).


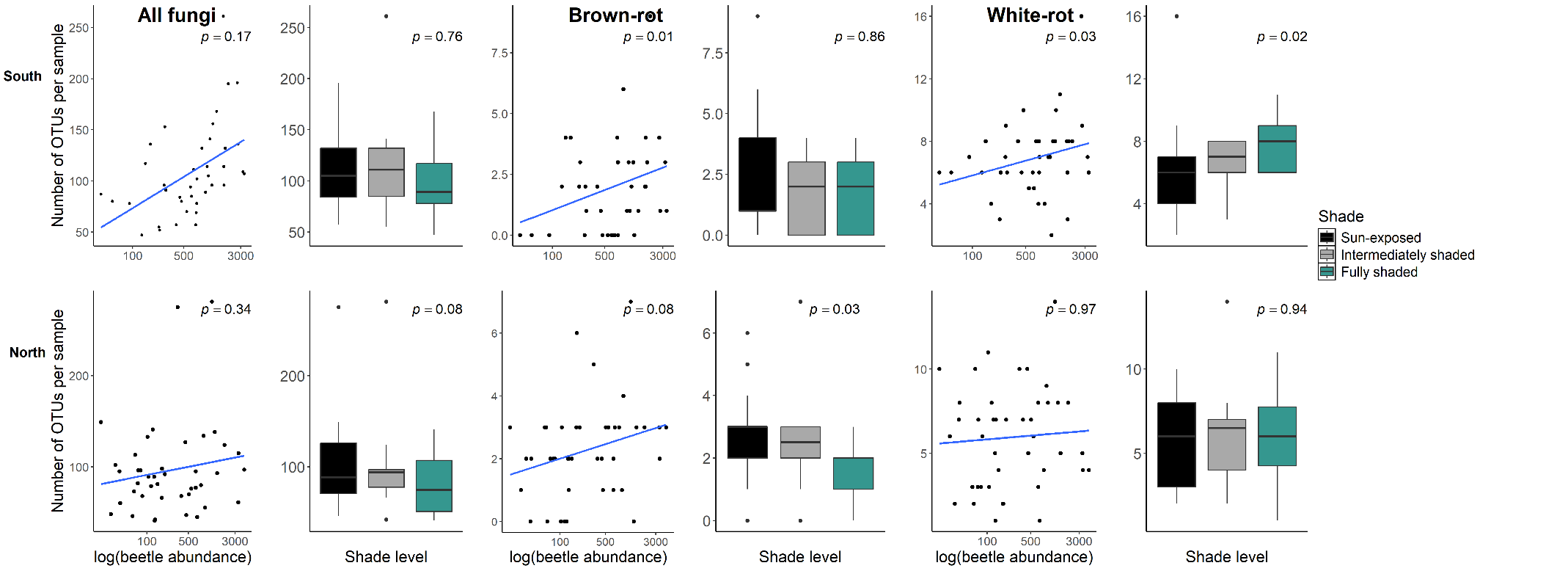


Figure S2. Dataset 2: Species richness in spruce logs of all fungi, brown-rotters, and white-rotters along a beetle abundance gradient (log-transformed), and along a shade gradient (respective boxplots to the right). The upper panels show the effects in the south (Tönnersjöheden, Asa, Siljansfors), and the lower panels the effects in the north (Vindeln, Järpen, Ätnarova).
